# Serum Albumin Levels and Economic Status in Japanese Older Adults

**DOI:** 10.1371/journal.pone.0155022

**Published:** 2016-06-08

**Authors:** Asami Ota, Naoki Kondo, Nobuko Murayama, Naohito Tanabe, Yugo Shobugawa, Katsunori Kondo, Japan Gerontological Evaluation Study (JAGES) group

**Affiliations:** 1 Division of Health and Nutrition, University of Niigata Prefecture, Niigata, Japan; 2 Department of Health and Social Behavior/Department of Health Education and Health Sociology, School of Public Health, The University of Tokyo, Tokyo, Japan; 3 Division of International Health, Niigata University Graduate School of Medical and Dental Sciences, Niigata, Japan; 4 Center for Preventive Medical Science, Chiba University, Chiba, Japan; 5 Center for Well-being and Society, Nihon Fukushi University, Aichi, Japan; 6 National Center for Geriatrics and Gerontology, Obu City, Aichi, Japan; National Cardiovascular Center Hospital, JAPAN

## Abstract

**Background:**

Low serum albumin levels are associated with aging and medical conditions such as cancer, liver dysfunction, inflammation, and malnutrition and might be an independent predictor of long-term mortality in healthy older populations. We tested the hypothesis that economic status is associated with serum albumin levels and explained by nutritional and health status in Japanese older adults.

**Design:**

We performed a cross-sectional analysis using data from the Japan Gerontological Evaluation study (JAGES). The study participants were 6528 functionally independent residents (3189 men and 3339 women) aged ≥65 years living in four municipalities in Aichi prefecture. We used household income as an indicator of economic status. Multiple linear regression was used to compare serum albumin levels in relation to household income, which was classified as low, middle, and high. Additionally, mediation by nutritional and health-related factors was analyzed in multivariable models.

**Results:**

With the middle-income group as reference, participants with low incomes had a significantly lower serum albumin level, even after adjustment for sex, age, residential area, education, marital status, and household structure. The estimated mean difference was −0.17 g/L (95% confidence interval, −0.33 to −0.01 g/L). The relation between serum albumin level and low income became statistically insignificant when “body mass index”, “consumption of meat or fish”, “self-rated health”, “presence of medical conditions”, “hyperlipidemia”, or “respiratory disease “was included in the model.

**Conclusion:**

Serum albumin levels were lower in Japanese older adults with low economic status. The decrease in albumin levels appears to be mediated by nutrition and health-related factors with low household incomes. Future studies are needed to reveal the existence of other pathways.

## Introduction

Japan is a rapidly aging society and has one of the oldest populations in the world; indeed, 25% of Japanese are older than 65 years. Average life expectancy at birth is 80.2 years for men and 86.6 years for women [1]. A government report noted that healthy life expectancy at birth is 71.2 years for men and 74.2 years for women, a 10-year difference from life expectancy values [2]. To extend the healthy life of older adults, identifying and addressing risk factors for health problems will be required [3–5].

All-cause mortality, cancer, functional disability, and mental illness, among many other medical conditions, are related to low socioeconomic status in adults and older adults [6–9]. The Annual Health, Labor and Welfare Report of 2014–2015 found that the number of welfare recipients has been consistently increasing in Japan since the end of the economic bubble, in the 1990s. Currently, 1.7% of Japanese receive government welfare support, and 45% of the recipients are older adults[10].

Serum albumin is the most abundant protein in plasma, and low serum albumin level might be a predictor of long-term mortality in general [11, 12] and elderly populations [13–17]. Decreased serum albumin is strongly associated with aging and reflects inflammation, frailty, and several pathological conditions, including cancer, rheumatoid arthritis, and liver dysfunction [18, 19]. Low economic status can impair health [4, 5] and nutrition status, and lower household expenditure is associated with a decrease in the quality of nutrient intake [20–22]; however, very few studies have focused on the association of serum albumin level and socioeconomic status [23, 24].

We hypothesized that low serum albumin level is an objective indicator of health and nutritional problems resulting from low economic status. Using baseline data from a cohort study, we investigated the relationship between serum albumin level and economic status (ie, household income) of Japanese older adults and assessed whether health and nutrition status mediated this relationship.

## Materials and Method

### Study participants

The present analysis is based on a data subset from the Japan Gerontological Evaluation Study (JAGES) 2010 project, a continuing prospective cohort study of factors associated with deteriorating health in adults aged 65 years or older. The sample was restricted to people with no baseline physical or cognitive disabilities, defined as receipt of public long-term care insurance benefits. The details of the project have been described elsewhere [6, 8, 25–29]. Briefly, self-administered postal questionnaires were distributed to a randomly sampled quarter of the population aged ≥65 years living in two municipalities (Tokai city, Chita city), and questionnaires were sent to all residents aged ≥65 years in two municipalities (Tokoname city and Taketoyo town) in Aichi prefecture. In 2010, 23.1% of the Japanese national population was aged 65 years or older. The percentages in the targeted communities were slightly lower: 15.7% in Tokai, 16.2% in Chita, 22.3% in Tokoname, and 15.6% in Taketoyo. Along with the questionnaire, we enclosed a written explanation of our use of health check-up data. Completion and return of the questionnaire was regarded as informed consent. Response rates in Tokai, Chita, Tokoname, and Taketoyo were 60.1%, 62.9%, 60.8%, and 61.1%, respectively.

Another dataset was obtained from annual health check-ups conducted by each municipality in 2010, in which more than 50% of elderly residents participated. During these health check-ups, height and weight were measured by staff at health centers, and body mass index (BMI) was calculated by dividing the body weight (in kilograms) by the height (in meters) squared. Blood samples were also collected and sent to each municipality’s health center or to hospitals, and serum albumin levels were measured with a colorimetric method.

From a total of 16,213 participants in the JAGES projects, 1968 were excluded because of missing information on age, sex, or household income. We also excluded 49 subjects who needed complete support for their activities of daily living. After linkage with health check-up data, 6528 functionally independent residents (3189 men and 3339 women) aged ≥65 years were ultimately included in the present analysis (S1 Appendix). The participants’ records were anonymized and de-identified before analysis. Ethical approval for the study was obtained from the Ethics Committee at Nihon Fukushi University.

### Measures

#### Household income

The JAGES 2010 questionnaire collected information on the total annual incomes of the participants’ household members in 15 predetermined categories (in thousands of yen). For each response, we calculated the equivalent household income by dividing income by the square root of the number of household members. From our previous studies [27–29], we divided our income variable into three categories as follows: low (10.2 to 158.8 thousand yen; n = 2794), middle (159.0 to 246.0 thousand yen; n = 1986), and high (247.5 to 919.2 thousand yen; n = 1748) income groups. The median incomes for these groups were 112.5 thousand yen, 190.2 thousand yen, and 317.6 thousand yen, respectively.

#### Nutritional factors

To investigate the interaction of nutritional factors, we analyzed BMI (from health checkup data) and frequency of meat/fish consumption (“meat/fish”) and frequency of vegetable consumption (“vegetables”) (from the JAGES questionnaire). We used BMI as the reference to determine balance of energy intake and consumption, as determined using the Dietary Reference Intakes for Japanese 2015 [30]. Because the recommended BMI for older adults is greater than 20 but less 25, we classified BMI into three groups: <20, 20 to <25, and ≥25. In the self-administered questionnaire for JAGES 2010, we asked, “How frequently do you eat fish or meat?” and “How frequently do you eat vegetables?”, and the answers were categorized as more than once per day, more than once per week, or less than once per week.

#### Health-related factors

The health-related factors assessed were self-rated health, activities of daily living, and presence of medical conditions. For self-rated health, we used the question, “How would you rate your overall health at the present time?” Four response options were provided: excellent, good, fair, or poor [31, 32]. For activities of daily living, we asked, “Do you need any support for your activities of daily living (walking, bathing, and toileting)?” The available response options were no need, some need, and total need. Individuals with a total need were excluded from the study.

Medical conditions were evaluated by asking whether individuals were currently receiving treatment for specific physical conditions, including cancer, heart disease, stroke, hypertension, diabetes mellitus, hyperlipidemia, respiratory disease, gastrointestinal disease, liver disease, and mental illness. Although some biomarkers (e.g., blood pressure, serum cholesterol, etc.) were simultaneously measured with serum albumin at the health check-up, we did not use those biomarkers as the covariates as single-time measurements were not equivalent to the diagnoses of the diseases.

#### Covariates

We included the following sociodemographic characteristics in the study: age group (65–70, 70–80, ≥80 years), sex, residential area (four municipalities), education (<high school [<10 years of education] or ≥high school [≥10years of education]), marital status (married, widowed, separated, or unmarried) and household structure (living alone, couple, three generations).

#### Statistical analysis

Summary statistics for numerical variables are expressed as mean ± standard deviation (SD). To compare characteristics of study participants in relation to household income, a multiple linear regression model was used to compare mean serum albumin levels, and multivariable multinomial regression models were used to compare categorical variables. In these models, serum albumin level and other categorical variables were treated as dependent variables, and dummy variables of household income groups were treated as independent variables. In every model, sex, age, and residential area were included as confounding variables to be controlled.

We created multiple linear regression models to examine associations between household income and serum albumin level. On the basis of regression coefficients and standard errors, mean difference (95% confidence interval [CI]) from the serum albumin level of the middle-income group (reference) was estimated in the other household income groups. Model 1 was adjusted for education (<10 years, ≥10 years, missing), marital status (married/widowed, separated, or unmarried/missing), and household structure (living alone, couple, three generations, missing). Then, nutritional and health-related factors were sequentially added to the Model 1. The categorical structures of these additional factors were similar to those described in Table 1. Mean differences in serum albumin levels between categories of each nutritional and health-related factor were also estimated by a model in which a single factor was added to model 1.

**Table 1:** Characteristic of participants.

Variable	Category	All		High Income		Middle Income		Low Income		P Value	P Value
Individuals(n = 7719)		n or mean ±SD	(%)	n or mean ±SD	(%)	n or mean ±SD	(%)	n or mean ±SD	(%)	unadjusted	adjusted ^a^
Serum albumin(g/L)		42.39±2.53		42.43±2.58		42.50±2.46		42.22±2.54		0.003	0.02
Sex	men	3189	48.9	1437	51.4	1003	50.5	749	42.8	<0.001	0.07
	Women	3339	51.1	1357	48.6	983	49.5	999	57.2		
Age (years)	65–69	2411	36.9	1104	39.5	775	39.0	532	30.4	<0.001	<0.001
	70–79	3385	51.9	1400	50.1	1021	51.4	964	55.1		
	80-	732	11.2	290	10.4	190	9.6	252	14.4		
Area	A	812	12.4	393	14.1	241	12.1	178	10.2	<0.001	0.52
	B	1340	20.5	621	22.2	394	19.8	325	18.6		
	C	2264	34.7	953	34.1	711	35.8	600	34.3		
	D	2112	32.4	827	29.6	640	32.2	645	36.9		
Education	<10	3104	47.5	1013	36.3	996	50.2	1095	62.6	<0.001	0.01
	≧10	3355	51.4	1757	62.9	974	49.0	624	35.7		
	missing	69	1.1	24	0.9	16	0.8	29	1.7		
Marital status	married	5223	80.0	2387	85.4	1564	78.8	1272	72.8	<0.001	0.01
	divorced, separated, never married	1250	19.1	397	14.2	407	20.5	446	25.5		
	missing	55	0.8	10	0.4	15	0.8	30	1.7		
Household structure	living alone	589	9.0	120	4.3	279	14.0	190	10.9	<0.001	<0.001
	couple	3006	46.0	1424	51.0	1064	53.6	518	29.6		
	three generations	2857	43.8	1218	43.6	615	31.0	1024	58.6		
	missing	76	1.2	32	1.1	28	1.4	16	0.9		

^a^ Adjusted for sex, age, and area.

A two-tailed P value of <0.05 was considered to indicate statistical significance. All analyses were performed using SPSS statistical software (version 17.0, SPSS, Chicago, IL, USA).

## Results

The mean age of the study participants was 72.4 ± 5.3 years, and the mean serum albumin level was 42.39 ± 2.53 g/L. Table 1 shows the characteristics of the participants in relation to household income. Serum albumin level significantly differed (P<0.003) in relation to household income. Participants with low incomes had the lowest level (42.22 ± 2.54 g/L). Sex, age group, area, education, marital status, and household structure also significantly differed among groups (P<0.001).

All nutritional factors except BMI significantly differed in relation to household income (Table 2). Older adults with lower incomes tended to eat meat/fish (P<0.001) and vegetables (P<0.001) less frequently. All health-related factors significantly differed among groups (P<0.001).

**Table 2:** Characteristics of study participants (nutritional and health factors).

Variable	Category	All		High Income		Middle Income		Low Income		P Value	P Value
		n or mean ±SD	(%)	n or mean ±SD	(%)	n or mean ±SD	(%)	n or mean ±SD	(%)	unadjusted	adjusted ^a^
**Nutritional Factors**											
**Body Mass Index(BMI)**	<20	1001	15.3	421	15.1	290	14.6	290	16.6	0.139	0.80
	20–25	3965	60.7	1720	61.6	1216	61.2	1029	58.9		
	≧25	1562	23.9	653	23.4	480	24.2	429	24.5		
**Frequency of eating meat or fish**	daily	2383	36.5	1212	43.4	661	33.3	510	29.2	<0.01	0.00
	1>/week	3299	50.5	1277	45.7	1075	54.1	947	54.2		
	≦1/week	381	5.8	162	5.8	107	5.4	112	6.4		
	missing	465	7.1	143	5.1	143	7.2	179	10.2		
**Frequency of eating vegetables**	daily	5053	77.4	2272	81.3	1508	75.9	1273	72.8	<0.01	0.27
	≦1/week	1067	16.3	368	13.2	355	17.9	344	19.7		
	≦1/week	56	0.9	14	0.5	19	1.0	23	1.3		
	missing	352	5.4	140	5.0	104	5.2	108	6.2		
**Health-related factors**											
**Self-rated health**	very good	739	11.3	347	12.4	208	10.5	184	10.5	<0.01	0.08
	good	4633	71.0	2028	72.6	1417	71.3	1188	68.0		
	bad	943	14.4	351	12.6	294	14.8	298	17.0		
	very bad	131	2.0	46	1.6	34	1.7	51	2.9		
	missing	82	1.3	22	0.8	33	1.7	27	1.5		
**Activities of daily living**	no need for help	6404	98.1	2765	99.0	1948	98.1	1691	96.7	<0.01	0.91
	needs partial help	45	0.7	10	0.4	13	0.7	22	1.3		
	missing	79	1.2	19	0.7	25	1.3	35	2.0		
**Treatment of any diseases**	yes	4572	70.0	1972	70.6	1397	70.3	1203	68.8	<0.01	0.10
	no	1520	23.3	650	23.3	478	24.1	392	22.4		
	missing	436	6.7	172	6.2	111	5.6	153	8.8		
**"Yes" for Treatment of any disease**											
**cancer**	yes	183	2.8	72	2.6	60	3.0	51	2.9	<0.01	0.08
	no	5909	90.5	2550	91.3	1815	91.4	1544	88.3		
	missing	436	6.7	172	6.2	111	5.6	153	8.8		
**heart disease**	yes	612	9.4	258	9.2	195	9.8	159	9.1	<0.01	0.08
	no	5480	83.9	2364	84.6	1680	84.6	1436	82.2		
	missing	436	6.7	172	6.2	111	5.6	153	8.8		
**stroke**	yes	67	1.0	28	1.0	14	0.7	25	1.4	<0.01	0.02
	no	6025	92.3	2594	92.8	1861	93.7	1570	89.8		
	missing	436	6.7	172	6.2	111	5.6	153	8.8		
**hypertension**	yes	2433	37.3	1041	37.3	719	36.2	673	38.5	<0.01	0.04
	no	3659	56.1	1581	56.6	1156	58.2	922	52.7		
	missing	436	6.7	172	6.2	111	5.6	153	8.8		
**diabetes**	yes	713	10.9	297	10.6	207	10.4	209	12.0	<0.01	0.05
	no	5379	82.4	2325	83.2	1668	84.0	1386	79.3		
	missing	436	6.7	172	6.2	111	5.6	153	8.8		
**hyperlipidemia**	yes	718	11.0	369	13.2	217	10.9	132	7.6	<0.01	0.10
	no	5374	82.3	2253	80.6	1658	83.5	1463	83.7		
	missing	436	6.7	172	6.2	111	5.6	153	8.8		
**arithritis**	yes	613	9.4	250	8.9	199	10.0	164	9.4	<0.01	0.04
	no	5479	83.9	2372	84.9	1676	84.4	1431	81.9		
	missing	436	6.7	172	6.2	111	5.6	153	8.8		
**respiratory disease**	yes	200	3.1	81	2.9	51	2.6	68	3.9	<0.01	0.03
	no	5892	90.3	2541	90.9	1824	91.8	1527	87.4		
	missing	436	6.7	172	6.2	111	5.6	153	8.8		
**gastric diseases**	yes	333	5.1	128	4.6	116	5.8	89	5.1	<0.01	0.02
	no	5759	88.2	2494	89.3	1759	88.6	1506	86.2		
	missing	436	6.7	172	6.2	111	5.6	153	8.8		
**liver disease**	yes	87	1.3	40	1.4	27	1.4	20	1.1	<0.01	0.10
	no	6005	92.0	2582	92.4	1848	93.1	1575	90.1		
	missing	436	6.7	172	6.2	111	5.6	153	8.8		
**mental illness**	yes	66	1.0	19	0.7	26	1.3	21	1.2	<0.01	0.10
	no	6026	92.3	2603	93.2	1849	93.1	1574	90.0		
	missing	436	6.6789216	172	6.2	111	5.6	153	8.8		

^a^Adjusted for sex, age, and area.

Table 3 shows associations between serum albumin level and household income. In the model adjusted for sex, age, and residential area, mean albumin level was significantly lower in older adults with low incomes (odds ratio, −0.19 g/L; 95% CI, −0.35 to −0.03 g/L), as compared with the middle-income group. The differences remained significant even after further adjustment for marital status, education, and family structure, in model 1. The mean difference in serum albumin level between the low-income and middle-income groups was −0.17 g/L (95% CI, −0.33 to −0.01 g/L).

**Table 3:** Association between serum albumin level and income level.

	High Income	Middle Income	Low Income
	Estimated mean difference from middle income.	reference	Estimated mean difference from middle income.
	-0.05(-0.19-0.09)	1	-0.19(-0.35--0.03)*
**Model1 ^a^**	-0.07(-0.21-0.08)	1	-0.17(-0.33--0.01)*
**BMI**	-0.07(-0.21-0.08)	1	-0.16(-0.32-0.01)
**meat/fish**	-0.07(-0.22-0.08)	1	-0.16(-0.32-0.01)
**vegetables**	-0.07(-0.21-0.08)	1	-0.17(-0.33--0.02)*
**self-rated health**	-0.08(-0.22-0.07)	1	-0.16(-0.32-0.01)
**activities of daily living**	-0.07(-0.21-0.08)	1	-0.17(-0.33--0.03)*
**Treatment of any diseases**	-0.07(-0.21-0.08)		-0.16(-0.33-0.03)
**"Yes" for treatment of any diseases^c^**			
**cancer**	-0.07(-0.21-0.08)	1	-0.16(-0.33-0.01)
**heart disease**	-0.07(-0.21-0.08)	1	-0.16(-0.33-0.00)
**stroke**	-0.07(-0.21-0.08)	1	-0.17(-0.30-0.00)
**hypertension**	-0.07(-0.22-0.08)	1	-0.18(-0.33-0.00)
**diabetes**	-0.07(-0.21-0.08)	1	-0.16(-0.32-0.01)
**hyperlipidemia**	-0.08(-0.23-0.06)	1	-0.14(-0.31-0.02)
**arthritis**	-0.07(-0.21-0.08)	1	-0.17(-0.33--0.04)*
**respiratory disease**	-0.06(-0.21-0.08)	1	-0.15(-0.32-0.01)
**gastrointestinal disease**	-0.07(-0.21-0.08)	1	-0.16(-0.33--0.01)*
**liver diseases**	-0.07(-0.21-0.08)	1	-0.16(-0.33-0.00)
**mental illness**	-0.07(-0.21-0.08)	1	-0.17(-0.34--0.01)*
**Model2 ^b^**	-0.07(-0.22-0.08)	1	-0.15(-0.31-0.02)
**Model3 ^d^**	-0.08(-0.22-0.07)	1	-0.15(-0.32-0.01)
**Model4 ^e^**	-0.08(-0.22-0.07)	1	-0.14(-0.30-0.03)

* P<0.05

^a^ Model 1 was adjusted for sex, age, area, education, marital status, and household structure.

^b^ Model 2 was adjusted for sex, age, area, education, marital status, household structure, BMI, frequency of meat or fish consumption, and frequency of vegetable consumption

^c^ Diseases under treatment were separately included in the model.

^d^ Model 3 was adjusted for sex, age, area, education, marital status, household structure, self-rated health, activities of daily living, and treatment for any disease.

^e^ Model 4 was adjusted for sex, age, area, education, marital status, household structure, BMI, frequency of meat or fish consumption, frequency of vegetable consumption, self-rated health, activities of daily living, and treatment for any disease.

When BMI was additionally included in model 1, the estimated mean difference in the low-income group decreased to −0.16 g/L (95% CI, −0.32 to 0.01 g/L), and when consumption of meat/fish was included it again decreased to −0.16 g/L (−0.32 to 0.01 g/L). These differences were not significant. In model 2, in which all three nutritional factors were included, the estimated mean difference further decreased, to −0.15 g/L (−0.31 to 0.02 g/L).

We then added the health-related factors to model 1. When self-rated health and treatment for any disease were included, the estimated mean differences for the low-income group became insignificant: −0.16 g/L (95% CI, −0.32 to 0.01 g/L) and −0.16 g/L (−0.33 to 0.03 g/L), respectively. When individual diseases under treatment were included in the model, the significance of the associations with low income disappeared for most diseases. A marked decrease in the estimated mean difference for the low-income group was seen for respiratory disease and hyperlipidemia (mean difference, −0.15 g/L [95% CI, −0.32 to 0.01 g/L] and −0.14 g/L [−0.31 to 0.02 g/L], respectively).

In model 3, which included all health-related factors, the estimated mean difference for the low-income group was −0.15 g/L (95% CI, −0.32 to 0.01 g/L). When all nutritional and health-related factors were simultaneously included in the model (model 4), the estimated mean difference for the low-income group decreased substantially, to −0.14 g/L (−0.30 to 0.03 g/L).

In the analysis of the associations of serum albumin with nutritional and health-related factors, BMI was strongly correlated with serum albumin. With daily consumption as reference, “eating meat/fish less than once per week” was significantly associated with lower serum albumin (Table 4). Among health-related factors, self-rated health was strongly correlated with serum albumin, and lower self-rated health was associated with low serum albumin level. As for disease status, older adults with respiratory and liver disease had lower serum albumin levels than did those with no disease, and serum albumin was higher in older adults with hypertension and hyperlipidemia.

**Table 4:** Associations of nutritional and health-related factors with serum albumin (g/L).

Variable	Category	Esitimated difference from reference category ^a^
**Body mass index**	<20	-0.49(-0.65 - -0.33)*
	20-25	1.00
	≧25	0.16(0.02 - 0.29)*
**Frequency of eating meat or fish**	daily	1.00
	1>/week	-0.02(-0.15 - 0.10)
	≦1/week	-0.27(-0.50 - -0.04)*
	missing	-0.01(-0.26 - 0.23)
**Frequency of eating vegetables**	daily	1.00
	1>/week	0.01(-0.14 - 0.17)
	≦1/week	-0.2(-0.78 - 0.39)
	missing	-0.02(-0.26 - 0.23)
**Activities of daily living**	no need for help	1.00
	need partial help	-0.02(-0.65 - 0.60)
	missing	-0.24(-0.67 - 0.19)
**Self-rated health**	very good	1.00
	good	-0.11(-0.29 - 0.07)
	bad	-0.39(-0.61- -0.16)*
	very bad	-0.51(-0.92- -0.10)*
	missing	-0.3(-0.79 - 0.19)
**Treatment of any diseases**	yes	0.09(-0.05 - 0.23)
	no	1.00
	missing	0.04(-0.20- 0.28)
**cancer**	yes	0.03(-0.31- 0.37)
	no	1.00
	missing	-0.03(-0.25 - 0.18)
**heart disease**	yes	-0.13(-0.32 - 0.06)
	no	1.00
	missing	-0.05(-0.26 - 0.17)
**stroke**	yes	0.39(-0.19 - 0.97)
	no	1.00
	missing	-0.03(-0.24 - 0.19)
**hypertension**	yes	0.28(0.16 - 0.40)*
	no	1.00
	missing	0.08(-0.14 - 0.30)
**diabetes**	yes	0.03(-0.15 - 0.22)
	no	1.00
	missing	-0.03(-0.24 - 0.19)
**hyperlipidemia**	yes	0.74(0.55 - 0.92)*
	no	1.00
	missing	0.04(-0.17 - 0.26)
**arthritis**	yes	-0.27(-0.45 - -0.08)*
	no	1.00
	missing	-0.06(-0.28 - 0.15)
**respiratory**	yes	-0.67(-1.01- -0.35)*
	no	1.00
	missing	-0.06(-0.27 - 0.16)
**gastrointestinal**	yes	0.01(-0.25 - 0.27)
	no	1.00
	missing	-0.03(-0.25 - 0.18)
**liver**	yes	-0.89(-1.37 - -0.40)*
	no	1.00
	missing	-0.04(-0.26 - 0.17)
**mental illness**	yes	-0.18(-0.77 - 0.40)
	no	1.00
	missing	0.09(-0.15 - 0.34)

* P<0.05

^a^ Adjusted for sex, age, area, equivalent income, education, marital status, and household structure.

## Discussion

To our knowledge, this is the first study to show a significant association between serum albumin and economic status among older adults in Japan or any other country. This cross-sectional study using baseline data from the JAGES project revealed that serum albumin level was significantly lower in a low-income group than in a middle-income group, even after adjustment for sex, age, area, education, marital status, and household structure. The statistical significance of lower serum albumin levels in the low-income group disappeared after adjustment for the confounding effects of nutrition and health-related factors, which suggests that these factors mediate the relationship.

Serum albumin is a biomarker that predicts all-cause mortality in general and elder populations [12, 13]. Studies showed that serum albumin independently predicted all-cause 10-year mortality in community-dwelling older adults [15, 16] or it could be a short-term predictor of mortality among institutionalized older adults [14]. Serum albumin is also an indicator of diseases such as cancer, rheumatoid arthritis, and liver dysfunction [18, 19] and of underlying health conditions such as inflammation, frailty, low activities of daily living score, cognitive decline, and malnutrition in older adults [26, 33–37]. These previous findings suggest that albumin might be a good measure of objective health status among community-dwelling older adults. Our results indicate that loss of healthy life is affected by economic status among community-dwelling Japanese older adults and that serum albumin might mediate these factors.

Very few studies have focused on serum albumin and socioeconomic status. In 2008, Kohler et al. reported an association between serum albumin and socioeconomic status (a composite of education, marital status, religion, wealth) in 906 participants in Malawi, but there was no association with any of the individual factors [23]. Louie et al. reported that serum albumin level was not associated with education level, poverty index, or race among participants aged 60 years or older [24]. Another study reported that race, country, and other background factors mediated associations between health-related biomarkers and socioeconomic status, and that stronger associations were seen in high-income countries than in middle- and low-income countries [38].

Nutrition and health-related factors might be pathways leading to low serum albumin levels in low-income older adults. Meat/fish consumption was a mediator of the relation between serum albumin and low income in the present study. A previous Japanese study showed that serum albumin was weakly positively associated with animal protein intake [39]. The present study found that serum albumin was significantly lower in income groups with a meat/fish consumption of less than once per week. Our study also demonstrated that, as compared with daily consumption, eating meat or fish less than once per week was significantly associated with low serum albumin. In studies of nutrition and socioeconomic status using nationally representative data from Japan, adults aged 18 to 74 years with higher household expenditures were likely to have a healthier, more favorable, nutrient intake [21]. These studies provide further support for our finding that meat/fish consumption mediated the relationship between serum albumin and socioeconomic status. Previous analyses of micronutrient intake and socioeconomic status yielded similar results: values for all macronutrients were lower for low socioeconomic status groups than for groups with high socioeconomic status [22].

Self-rated health, treatment of any disease, and presence of diseases such as respiratory disease and hyperlipidemia were intermediate factors in the relation of serum albumin with economic status in the low-income group. Like serum albumin, self-rated health predicts subsequent mortality and is used as a measure of general health [31, 32]. Activities of daily living did not mediate this effect, perhaps because older adults with low activities of daily living scores were excluded from the study.

Among diseases under treatment assessed in the present study, respiratory disease was previously reported to be an important cause of death in older adults with a low BMI [40–42]. Our results showed a similar pattern, namely, an association between presence of respiratory disease and low albumin level. Like BMI, cholesterol levels tend to correlate with serum albumin, and low cholesterol is related to high mortality, diminished health status, underlying inflammation, and under nutrition [43, 44]. Thus, low-income older adults were less likely than middle-income older adults to receive a diagnosis of hyperlipidemia in the study.

These findings suggest that health status and certain diseases are related to albumin level and economic status. Our findings suggest that other diseases, such as cancer, heart disease, stroke, hypertension, diabetes, and liver disease, might also mediate the relation between serum albumin and socioeconomic status. Higher mortality from cancer and stroke has been reported in Japanese with low socioeconomic status, [45–47] and future studies should attempt to clarify the relationship between socioeconomic status and the incidences of these diseases.

In a study of the relations between health, nutrition, and socioeconomic status, Katsarou et al. found that among healthy adults aged 65–100 years living on a Mediterranean island, those with the highest socioeconomic status consumed more fish and vegetables than did those with the lowest socioeconomic status. Additionally, associations with dietary habits are mediated by the presence of chronic vascular disease factors such as hypertension, hypercholesterolemia, and diabetes [20]. An advantage of our approach, the use of serum albumin as a biomarker to examine relationships between health, nutrition, and socioeconomic status, is that serum albumin is an objective indicator that might assist in assessing elder health.

The nutritional questionnaire used in the JAGES 2010 study asked only about fish/meat consumption. A previous study showed that in younger and older adults dietary protein consumption and quantity affects serum albumin level[48]. In a Japanese study, animal protein intake was related to serum albumin level, although vegetable protein intake was not[39]. In future studies, questions on detailed food groups, such as eggs, beans, and milk, might need to be added to determine how low-income older adults transition to poor nutrition and health, and how such transitions can be prevented. There might be other bridging factors between low serum albumin level and socioeconomic status.

Sahyoun et al. found that increased mortality in persons with low serum albumin levels was associated with age, but also with history of chronic diseases, protein intake, and other factors that could not be evaluated in their study[14]. Serum albumin may bridge geriatric physical and mental problems in conditions such as frailty, impaired activities of daily living, cognitive decline, and malnutrition [33–37, 49]. Low serum albumin in the present older adults with low incomes might reflect not only health and nutrition factors but also factors that could not be investigated in our study.

Our study has a number of limitations. First, the response rate to our baseline invitation was only 60%, which decreased to 50% when respondents with incomplete health checkup data were excluded. Second, selection bias is a concern, as we used data from health checkups, which might include information from older adults who were more health-conscious. Still, if an association between serum albumin and income is present in relatively healthy people, health disparities in relation to economic status are likely. Third, because cross-sectional studies cannot establish causality, a longitudinal study might be needed in order to analyze changes in annual income and serum albumin, to exclude the possibility of reverse causation that health problems and decreased serum albumin resulted in low incomes. Finally, disease information was self-reported, potentially causing reporting bias.

The results of this study have important public health implications. Among Japanese older adults, those with low incomes tend to have lower serum albumin levels. This supports the results of past studies, which show that low economic status is associated with worsening health among older adults. We also found evidence that protein intake in older adults might be an interacting factor between serum albumin and income level. Thus, improving intake of good food—with food vouchers or a reduction or waiver of taxes on food—might decrease mortality and improve health among older adults.

The relation of respiratory disease to serum albumin and economic status and the pathway from low income to respiratory disease need to be acknowledged and addressed. In Japan, pneumonia is the third leading cause of death and chronic obstructive pulmonary disease is the 10th leading cause [50]. Fukuda et al. reported that the rate of current smoking increased with decreasing household expenditure [51]. Antismoking measures for older adults, especially those with low socioeconomic status, might help prevent deaths from respiratory diseases. Future studies should examine the pathways by which low socioeconomic status leads to poor health and disease development.

In conclusion, we found a significant association between serum albumin level and household income among community-dwelling Japanese elders. Population aging is an important reason for the increasing inequalities in income and health in Japanese society. Thus, it is necessary to evaluate and monitor overall health status in healthy community-dwelling older adults, to ensure prompt intervention, when necessary. The evidence suggests that the association between serum albumin level and household income is mediated by health-related and nutritional factors among low-income older adults.

## Supporting Information

S1 Appendix Flowchart of participants included in the study. (PDF)

## References

[1] World health statistics 2014. World Health Organization.

[2] Shuji Hashimoto KM, Toshiyuki Ojima. Index of Healthy life expectancy in Japan. In: Ministry of Health LaW, editor. 2014.

[3] Marmot M, Allen J, Bell R, Bloomer E, Goldblatt P. WHO European review of social determinants of health and the health divide. Lancet (London, England). 2012;380(9846):1011–29. Epub 2012/09/12. 10.1016/s0140-6736(12)61228-8 . 22964159

[4] Smith GD. Learning to live with complexity: ethnicity, socioeconomic position, and health in Britain and the United States. American journal of public health. 2000;90(11):1694–8. Epub 2000/11/15. ; PubMed Central PMCID: PMCPmc1446401. 11076232 10.2105/ajph.90.11.1694 PMC1446401

[5] Kagamimori S, Gaina A, Nasermoaddeli A. Socioeconomic status and health in the Japanese population. Social science & medicine. 2009;68(12):2152–60. 10.1016/j.socscimed.2009.03.030 . 19375838

[6] Ichida Y, Kondo K, Hirai H, Hanibuchi T, Yoshikawa G, Murata C. Social capital, income inequality and self-rated health in Chita peninsula, Japan: a multilevel analysis of older people in 25 communities. Social science & medicine. 2009;69(4):489–99. 10.1016/j.socscimed.2009.05.006 . 19523728

[7] Fujino Y, Tamakoshi A, Iso H, Inaba Y, Kubo T, Ide R, et al A nationwide cohort study of educational background and major causes of death among the elderly population in Japan. Preventive medicine. 2005;40(4):444–51. Epub 2004/11/09. 10.1016/j.ypmed.2004.07.002 . 15530597

[8] Kondo N, Kawachi I, Hirai H, Kondo K, Subramanian SV, Hanibuchi T, et al Relative deprivation and incident functional disability among older Japanese women and men: prospective cohort study. Journal of epidemiology and community health. 2009;63(6):461–7. 10.1136/jech.2008.078642 19218252 PMC2677290

[9] Murata C, Kondo K, Hirai H, Ichida Y, Ojima T. Association between depression and socio-economic status among community-dwelling elderly in Japan: the Aichi Gerontological Evaluation Study (AGES). Health Place. 2008;14(3):406–14. Epub 2007/10/05. 10.1016/j.healthplace.2007.08.007 . 17913562

[10] Annual Report on Health, Labor and Welfare 2015. In: Ministry of Health LaW, editor. 2015.

[11] Phillips A, Shaper AG, Whincup PH. Association between serum albumin and mortality from cardiovascular disease, cancer, and other causes. Lancet (London, England). 1989;2(8677):1434–6. Epub 1989/12/16. . 2574367 10.1016/s0140-6736(89)92042-4

[12] Schalk BW, Visser M, Bremmer MA, Penninx BW, Bouter LM, Deeg DJ. Change of serum albumin and risk of cardiovascular disease and all-cause mortality: Longitudinal Aging Study Amsterdam. American journal of epidemiology. 2006;164(10):969–77. 10.1093/aje/kwj312 . 16980573

[13] Corti MC, Guralnik JM, Salive ME, Sorkin JD. Serum albumin level and physical disability as predictors of mortality in older persons. JAMA. 1994;272(13):1036–42. Epub 1994/10/05. . 8089886

[14] Sahyoun NR, Jacques PF, Dallal G, Russell RM. Use of albumin as a predictor of mortality in community dwelling and institutionalized elderly populations. Journal of clinical epidemiology. 1996;49(9):981–8. Epub 1996/09/01. . 8780605 10.1016/0895-4356(96)00135-7

[15] Takata Y, Ansai T, Yoshihara A, Miyazaki H. Serum albumin (SA) levels and 10-year mortality in a community-dwelling 70-year-old population. Archives of gerontology and geriatrics. 2012;54(1):39–43. 10.1016/j.archger.2011.02.018 . 21458870

[16] Shibata H, Haga H, Nagai H, Suyama Y, Yasumura S, Koyano W, et al Predictors of all-cause mortality between ages 70 and 80: the Koganei study. Archives of gerontology and geriatrics. 1992;14(3):283–97. Epub 1992/05/01. . 15374392 10.1016/0167-4943(92)90028-3

[17] Higashiguchi M, Nakaya N, Ohmori K, Shimazu T, Sone T, Hozawa A, et al [Malnutrition and the risk of long-term care insurance certification or mortality. A cohort study of the Tsurugaya project]. [Nihon koshu eisei zasshi] Japanese journal of public health. 2008;55(7):433–9. Epub 2008/09/04. . 18763618

[18] Fanali G, di Masi A, Trezza V, Marino M, Fasano M, Ascenzi P. Human serum albumin: from bench to bedside. Molecular aspects of medicine. 2012;33(3):209–90. 10.1016/j.mam.2011.12.002 . 22230555

[19] Don BR, Kaysen G. Serum albumin: relationship to inflammation and nutrition. Seminars in dialysis. 2004;17(6):432–7. Epub 2005/01/22. 10.1111/j.0894-0959.2004.17603.x . 15660573

[20] Katsarou A, Tyrovolas S, Psaltopoulou T, Zeimbekis A, Tsakountakis N, Bountziouka V, et al Socio-economic status, place of residence and dietary habits among the elderly: the Mediterranean islands study. Public health nutrition. 2010;13(10):1614–21. Epub 2010/04/01. 10.1017/s1368980010000479 . 20353616

[21] Fukuda Y. High quality nutrient intake is associated with higher household expenditures by Japanese adults. BioScience Trends. 2012;6(4):176–82. 10.5582/bst.2012.v6.4.176 23006964

[22] Novakovic R, Cavelaars A, Geelen A, Nikolic M, Altaba II, Vinas BR, et al Socio-economic determinants of micronutrient intake and status in Europe: a systematic review. Public health nutrition. 2014;17(5):1031–45. Epub 2013/06/12. 10.1017/s1368980013001341 . 23750829 PMC10282449

[23] Kohler IV, Soldo BJ, Anglewicz P, Chilima B, Kohler HP. Association of blood lipids, creatinine, albumin, and CRP with socioeconomic status in Malawi. Population health metrics. 2013;11(1):4 Epub 2013/03/02. 10.1186/1478-7954-11-4 ; PubMed Central PMCID: PMCPmc3701600. 23448548 PMC3701600

[24] Louie GH, Ward MM. Socioeconomic and ethnic differences in disease burden and disparities in physical function in older adults. American journal of public health. 2011;101(7):1322–9. 10.2105/AJPH.2010.199455 21164082 PMC3110229

[25] Ichida Y, Hirai H, Kondo K, Kawachi I, Takeda T, Endo H. Does social participation improve self-rated health in the older population? A quasi-experimental intervention study. Social science & medicine. 2013;94:83–90. Epub 2013/08/13. 10.1016/j.socscimed.2013.05.006 . 23931949

[26] Nishi A, Kawachi I, Shirai K, Hirai H, Jeong S, Kondo K. Sex/gender and socioeconomic differences in the predictive ability of self-rated health for mortality. PloS one. 2012;7(1):e30179 10.1371/journal.pone.0030179 22276157 PMC3261899

[27] Kanamori S, Kai Y, Aida J, Kondo K, Kawachi I, Hirai H, et al Social participation and the prevention of functional disability in older Japanese: the JAGES cohort study. PloS one. 2014;9(6):e99638 Epub 2014/06/14. 10.1371/journal.pone.0099638 ; PubMed Central PMCID: PMCPmc4055714. 24923270 PMC4055714

[28] Yamakita M, Kanamori S, Kondo N, Kondo K. Correlates of Regular Participation in Sports Groups among Japanese Older Adults: JAGES Cross-Sectional Study. PloS one. 2015;10(10):e0141638 Epub 2015/10/30. 10.1371/journal.pone.0141638 ; PubMed Central PMCID: PMCPmc4626107. 26512895 PMC4626107

[29] Hikichi H, Kondo N, Kondo K, Aida J, Takeda T, Kawachi I. Effect of a community intervention programme promoting social interactions on functional disability prevention for older adults: propensity score matching and instrumental variable analyses, JAGES Taketoyo study. Journal of epidemiology and community health. 2015;69(9):905–10. Epub 2015/04/19. 10.1136/jech-2014-205345 ; PubMed Central PMCID: PMCPmc4552922. 25888596 PMC4552922

[30] Hishida Akira SS. Overview of Dietary Reference Intakes for Japanese (2015). In: Ministry of Health LaW, editor. 2015.

[31] Salomon JA, Tandon A, Murray CJ. Comparability of self rated health: cross sectional multi-country survey using anchoring vignettes. Bmj. 2004;328(7434):258 10.1136/bmj.37963.691632.44 14742348 PMC324453

[32] Jylha M. What is self-rated health and why does it predict mortality? Towards a unified conceptual model. Social science & medicine. 2009;69(3):307–16. Epub 2009/06/13. 10.1016/j.socscimed.2009.05.013 . 19520474

[33] Kitamura K, Nakamura K, Nishiwaki T, Ueno K, Nakazawa A, Hasegawa M. Determination of whether the association between serum albumin and activities of daily living in frail elderly people is causal. Environmental health and preventive medicine. 2012;17(2):164–8. 10.1007/s12199-011-0233-y 21861116 PMC3342638

[34] Onem Y, Terekeci H, Kucukardali Y, Sahan B, Solmazgul E, Senol MG, et al Albumin, hemoglobin, body mass index, cognitive and functional performance in elderly persons living in nursing homes. Archives of gerontology and geriatrics. 2010;50(1):56–9. Epub 2009/02/24. 10.1016/j.archger.2009.01.010 . 19233487

[35] Taniguchi Y, Shinkai S, Nishi M, Murayama H, Nofuji Y, Yoshida H, et al Nutritional biomarkers and subsequent cognitive decline among community-dwelling older Japanese: a prospective study. J Gerontol A Biol Sci Med Sci. 2014;69(10):1276–83. Epub 2014/02/04. 10.1093/gerona/glt286 . 24488214

[36] Cabrerizo S, Cuadras D, Gomez-Busto F, Artaza-Artabe I, Marin-Ciancas F, Malafarina V. Serum albumin and health in older people: Review and meta analysis. Maturitas. 2015;81(1):17–27. Epub 2015/03/19. 10.1016/j.maturitas.2015.02.009 . 25782627

[37] Ng TP, Niti M, Feng L, Kua EH, Yap KB. Albumin, apolipoprotein E-epsilon4 and cognitive decline in community-dwelling Chinese older adults. Journal of the American Geriatrics Society. 2009;57(1):101–6. 10.1111/j.1532-5415.2008.02086.x . 19054180

[38] Goldman N, Turra CM, Rosero-Bixby L, Weir D, Crimmins E. Do biological measures mediate the relationship between education and health: A comparative study. Social science & medicine. 2011;72(2):307–15. 10.1016/j.socscimed.2010.11.004 21159415 PMC3039215

[39] Watanabe M, Higashiyama A, Kokubo Y, Ono Y, Okayama A, Okamura T. Protein Intakes and Serum Albumin Levels in a Japanese General Population: NIPPON DATA90. Journal of Epidemiology. 2010;20(Supplement_III):S531–S6. 10.2188/jea.JE20090221 20351474 PMC3920390

[40] Kvamme JM, Holmen J, Wilsgaard T, Florholmen J, Midthjell K, Jacobsen BK. Body mass index and mortality in elderly men and women: the Tromso and HUNT studies. J Epidemiol Community Health. 2012;66(7):611–7. Epub 2011/02/16. 10.1136/jech.2010.123232 21321065 PMC3368492

[41] Hong S, Yi SW, Sull JW, Hong JS, Jee SH, Ohrr H. Body mass index and mortality among Korean elderly in rural communities: Kangwha Cohort Study. PLoS One. 2015;10(2):e0117731 Epub 2015/02/27. 10.1371/journal.pone.0117731 25719567 PMC4342154

[42] Takata Y, Ansai T, Soh I, Awano S, Nakamichi I, Akifusa S, et al Body mass index and disease-specific mortality in an 80-year-old population at the 12-year follow-up. Arch Gerontol Geriatr. 2013;57(1):46–53. Epub 2013/03/13. 10.1016/j.archger.2013.02.006 . 23478161

[43] Reuben DB, Ix JH, Greendale GA, Seeman TE. The predictive value of combined hypoalbuminemia and hypocholesterolemia in high functioning community-dwelling older persons: MacArthur Studies of Successful Aging. J Am Geriatr Soc. 1999;47(4):402–6. Epub 1999/04/15. . 10203113 10.1111/j.1532-5415.1999.tb07230.x

[44] Jacobs D, Blackburn H, Higgins M, Reed D, Iso H, McMillan G, et al Report of the Conference on Low Blood Cholesterol: Mortality Associations. Circulation. 1992;86(3):1046–60. Epub 1992/09/01. . 1355411 10.1161/01.cir.86.3.1046

[45] Ueda K, Tsukuma H, Ajiki W, Oshima A. Socioeconomic factors and cancer incidence, mortality, and survival in a metropolitan area of Japan: a cross-sectional ecological study. Cancer science. 2005;96(10):684–8. Epub 2005/10/20. 10.1111/j.1349-7006.2005.00104.x . 16232200 PMC11158515

[46] Fukuda Y, Nakamura K, Takano T. Cause-specific mortality differences across socioeconomic position of municipalities in Japan, 1973–1977 and 1993–1998: increased importance of injury and suicide in inequality for ages under 75. International journal of epidemiology. 2005;34(1):100–9. Epub 2004/11/25. 10.1093/ije/dyh283 . 15561754

[47] Fujino Y, Iso H, Tamakoshi A, Inaba Y, Koizumi A, Kubo T, et al A Prospective Cohort Study of Employment Status and Mortality from Circulatory Disorders among Japanese Workers. Journal of occupational health. 2005;47(6):510–7. Epub 2005/12/22. . 16369114 10.1539/joh.47.510

[48] Thalacker-Mercer AE, Campbell WW. Dietary protein intake affects albumin fractional synthesis rate in younger and older adults equally. Nutrition reviews. 2008;66(2):91–5. Epub 2008/02/08. 10.1111/j.1753-4887.2007.00012.x . 18254875

[49] Vanitallie TB. Frailty in the elderly: contributions of sarcopenia and visceral protein depletion. Metabolism. 2003;52(10 Suppl 2):22–6. Epub 2003/10/25. . 14577059 10.1016/s0026-0495(03)00297-x

[50] Outline of Vital Statistics 2013. Ministry of Health, Labour and Welfare

[51] Fukuda Y, Hiyoshi A. Associations of household expenditure and marital status with cardiovascular risk factors in Japanese adults: analysis of nationally representative surveys. Journal of epidemiology / Japan Epidemiological Association. 2013;23(1):21–7. Epub 2012/12/05. ; PubMed Central PMCID: PMCPmc3700239. 23208515 10.2188/jea.JE20120021 PMC3700239

