# Supplementary material for: Serum Albumin Levels and Economic Status in Japanese Older Adults
Source: PLoS One. 2016 Jun 8;11(6):e0155022. doi: 10.1371/journal.pone.0155022 (PMC4898757; doi:10.1371/journal.pone.0155022)
Supplement: S1 Appendix — (PDF) [file pone.0155022.s001.pdf]

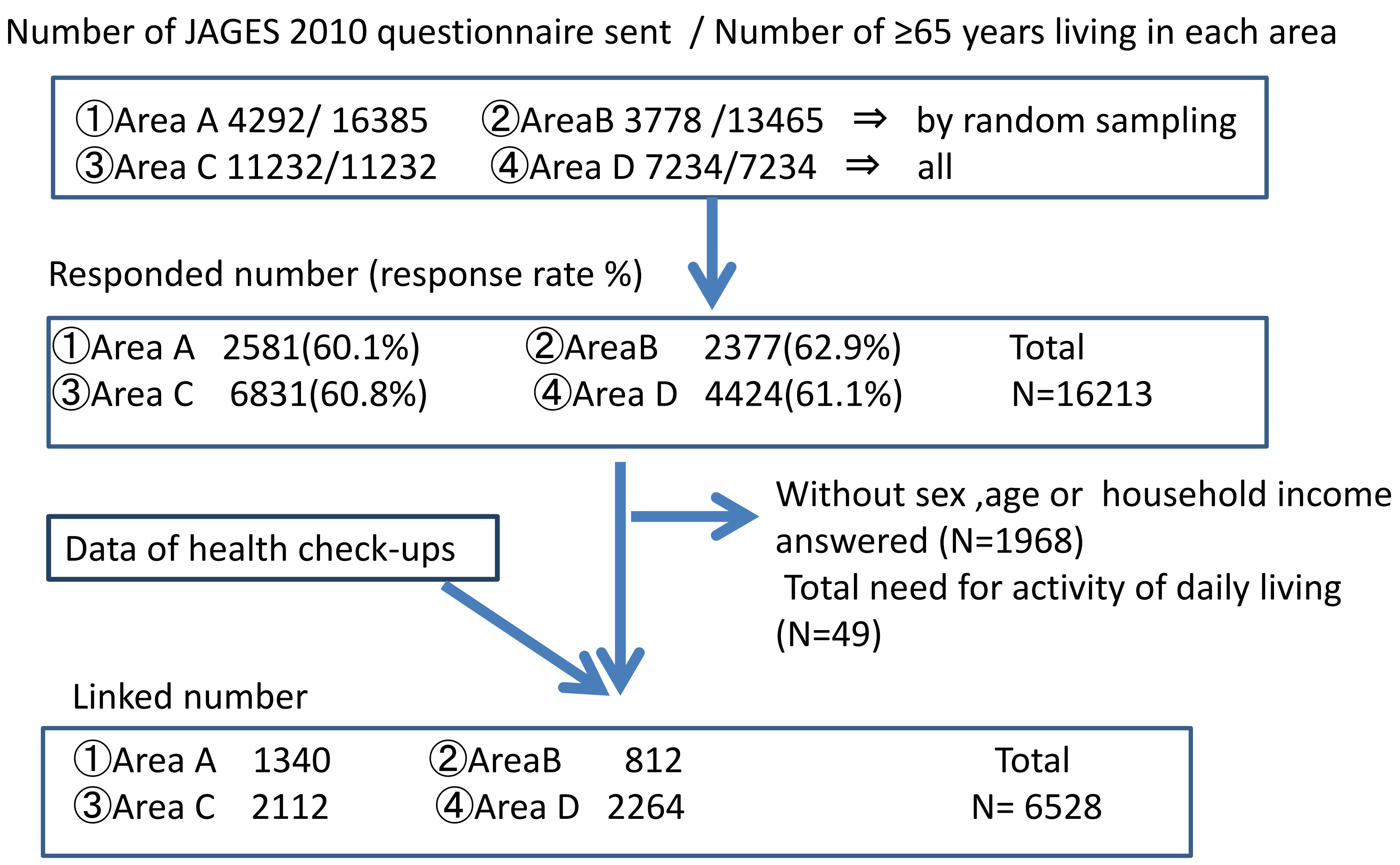
Number of JAGES 2010 questionnaire sent / Number of ≥65 years living in each area
①Area A 4292/ 16385 ②AreaB 3778 /13465 ⇒ by random sampling
③Area C 11232/11232 ④Area D 7234/7234 ⇒ all
Responded number (response rate %)
①Area A 2581(60.1%) ②AreaB 2377(62.9%) Total
③Area C 6831(60.8%) ④Area D 4424(61.1%) N=16213
Data of health check-ups
Without sex ,age or household income answered (N=1968)
Total need for activity of daily living (N=49)
Linked number
①Area A 1340 ②AreaB 812 Total
③Area C 2112 ④Area D 2264 N= 6528
